# Effects of Acupuncture on the Recovery Outcomes of Stroke Survivors with Shoulder Pain: A Systematic Review

**DOI:** 10.3389/fneur.2018.00030

**Published:** 2018-01-31

**Authors:** Janita Pak Chun Chau, Suzanne Hoi Shan Lo, Xingfeng Yu, Kai Chow Choi, Alexander Yuk Lun Lau, Justin Che Yuen Wu, Vivian Wing Yan Lee, William Hoi Ngai Cheung, Jessica Yuet Ling Ching, David R. Thompson

**Affiliations:** ^1^The Nethersole School of Nursing, Faculty of Medicine, The Chinese University of Hong Kong, Hong Kong, Hong Kong; ^2^Department of Medicine and Therapeutics, Faculty of Medicine, The Chinese University of Hong Kong, Hong Kong, Hong Kong; ^3^Hong Kong Institute of Integrative Medicine, The Chinese University of Hong Kong, Hong Kong, Hong Kong; ^4^School of Pharmacy, Faculty of Medicine, The Chinese University of Hong Kong, Hong Kong, Hong Kong; ^5^School of Nursing and Midwifery, Queen’s University Belfast, Belfast, United Kingdom

**Keywords:** stroke, rehabilitation, acupuncture, alternative and complementary medicine, poststroke shoulder pain, traditional Chinese medicine, systematic review

## Abstract

**Background:**

Poststroke shoulder pain limits stroke survivors’ physical functioning, impairs their ability to perform daily activities, and compromises their quality of life. The use of acupuncture to manage shoulder pain after a stroke is believed to free the blockage of energy flow and produce analgesic effects, but the evidence is unclear. We therefore conducted a systematic review to summarize the current evidence on the effects of acupuncture on the recovery outcomes of stroke survivors with shoulder pain.

**Methods:**

Fourteen English and Chinese databases were searched for data from January 2009 to August 2017. The review included adult participants with a clinical diagnosis of ischemic or hemorrhagic stroke who had developed shoulder pain and had undergone conventional acupuncture, electroacupuncture, fire needle acupuncture, or warm needle acupuncture. The participants in the comparison group received the usual stroke care only.

**Results:**

Twenty-nine randomized controlled trials were included. Most studies were assessed as having a substantial risk of bias. Moreover, due to the high heterogeneity of the acupuncture therapies examined, pooling the results in a meta-analysis was not appropriate. A narrative summary of the results is thus presented. The review showed that conventional acupuncture can be associated with benefits in reducing pain and edema and improving upper extremity function and physical function. The effects of conventional acupuncture on improving shoulder range of motion (ROM) are in doubt because this outcome was only examined in two trials. Electroacupuncture might be effective in reducing shoulder pain and improving upper extremity function, and conclusions on the effects of electroacupuncture on edema, shoulder ROM, and physical function cannot be drawn due to the limited number of eligible trials. The evidence to support the use of fire needle or warm needle acupuncture in stroke survivors with shoulder pain is also inconclusive due to the limited number of studies.

**Conclusion:**

Although most studies reviewed concluded that conventional and electroacupuncture could be effective for management of shoulder pain after stroke, the very high potential for bias should be considered. Further work in this area is needed that employs standardized acupuncture treatment modalities, endpoint assessments, and blinding of treatments.

## Introduction

Shoulder pain is a disabling complication after stroke. Its prevalence ranges from 54 to 75% in the first 6–12 months after a stroke (1, 2). A multicenter prospective study (3) reported a prevalence of 14% in the acute stroke stage and 43% and 32% in the subacute and chronic stroke stages, respectively, among 546 stroke survivors. It has been claimed that once shoulder pain has developed, survivors have a higher risk of persistent shoulder pain for several months or even a year afterward (4, 5).

Poststroke shoulder pain (PSP) may result from a brain lesion, altered neuromuscular control, or prior damage in the shoulder area, such as adhesive capsulitis and rotator cuff disorders (6–8). Prolonged immobility or improper positioning that cause repetitive trauma to the shoulder are also associated with PSP (5). The experience of pain limits stroke survivors’ range of motion (ROM) and motor functioning of the affected shoulder (9). It also impairs their ability to participate in daily or social activities and further compromises their health-related quality of life (10). More importantly, stroke survivors may have poorer motivation to pursue rehabilitation training due to intolerable pain, which, in turn, may have a negative effect on their recovery outcomes (1).

Typical interventions for PSP range from neuromuscular or transcutaneous electrical nerve stimulation of motor or sensory nerves for pain relief, application of supportive devices such as tape, to pharmacological therapy, including botulinum toxin, subacromial or intraarticular glenohumeral corticosteroid injections, or nerve blockers (1). Nevertheless, there is inconclusive evidence for the effectiveness of shoulder taping in reducing PSP (11–13). Manual techniques such as positioning, stretching, ROM exercises, or massage likely promote comfort only due to the lack of empirical evidence for their effects (1, 14).

Evidence suggests that acupuncture has potential benefits for individuals with PSP (1). Acupuncture has been practiced for more than 2,500 years as an important therapeutic technique of traditional Chinese medicine (TCM) for the treatment of various health problems, including pain, headache, musculoskeletal disorders, and psychological problems. The systematic theory of acupuncture was well established and documented in the Yellow Emperor’s Classic of Internal Medicine (15). In recent decades, with the growing popularity of TCM worldwide, the effects of acupuncture for ameliorating PSP have been intensively investigated.

The first systematic review conducted in 2009 on the effects of acupuncture on PSP included 7 randomized controlled trials (RCTs) and 502 stroke survivors (16). A narrative summary of the findings was presented, and no single estimate of the effects of acupuncture was provided. A second systematic review with meta-analysis on the same topic included 12 trials and 1,002 participants (17). Both reviews found that acupuncture was associated with potential benefits in reducing PSP. Empirical evidence of the effects of acupuncture for survivors with PSP is emerging rapidly. Therefore, the purpose of this systematic review is to update the summaries of such evidence. We reviewed and synthesized all available evidence up to August 2017 regarding the beneficial effects of various types of acupuncture on recovery outcomes, among which shoulder pain is the primary outcome, of survivors with PSP.

## Materials and Methods

The review adhered to the preferred reporting items for systematic reviews and meta-analysis guidelines (Data Sheet S1 in Supplementary Material) (18).

### Criteria of Eligibility

#### Population

We included trials that recruited adult participants (18 years of age or above) with a clinical diagnosis of ischemic or hemorrhagic stroke (19) who had shoulder pain [e.g., hemiplegic shoulder pain (HSP) or shoulder–hand syndrome developed following a stroke], regardless of the duration after stroke onset, severity of stroke, and level of physical function at the time acupuncture was received. HSP and PSP are often used interchangeably, but not all survivors with HSP had hemiplegia (20). Shoulder–hand syndrome is also referred to as complex regional pain syndrome (21). We excluded trials that recruited participants with an underlying fracture or shoulder dislocation.

#### Interventions

The participants in the intervention groups of these trials had to have received acupuncture targeting survivors with PSP on the basis of comparator interventions. Conventional acupuncture included traditional acupuncture (i.e., insertion of an acupuncture needle into acupoints) and balance acupuncture (i.e., insertion of an acupuncture needle into the peripheral nerves) (22). The underpinning mechanism of balance acupuncture is to promote the self-recovery of clients by stimulating the peripheral nerves by manipulation of the central nervous system (22). Electroacupuncture is the application of electrical stimulation to the inserted needles (23). Fire needle acupuncture is a treatment in which the acupuncture needles are heated before insertion into the acupoints. Warm needle acupuncture is a treatment that combines traditional acupuncture with moxibustion in which a small amount of moxa is placed on the acupuncture needle after insertion.

The practitioners who delivered the acupuncture therapies could use a fixed set of acupoints or a flexible combination of acupoints, depending on the symptoms and the extent of PSP. We considered acupuncture therapies that used all sizes and types of acupuncture needles, any frequency and duration of needle retention, and any number and location of acupoints.

#### Comparison

The participants in the comparison group received usual stroke care only, ranging from stroke rehabilitation programs to promote lifestyle changes, rehabilitation exercises to improve physical function, positioning, and the use of herbal medicine, to treat stroke and reduce shoulder pain (24, 25).

#### Outcomes of Interest

The primary outcome was shoulder pain, and the secondary outcomes included upper extremity function, edema, shoulder ROM, physical function, health-related quality of life, depression, self-esteem, and adverse events related to the acupuncture therapies received. We included all measurement time points examined in the included trials.

#### Studies

The review included only RCTs with a parallel or crossover design published in English or Chinese. Theses and conference papers were excluded. Trials that compared the effectiveness of different acupuncture therapies were excluded. Trials were also excluded if important intervention details or outcomes were not available despite attempts to contact the authors.

### Information Sources

Six English electronic bibliographic databases—MEDLINE, EMBASE, PsycINFO, CINAHL, AMED, and the Cochrane Central Register of Controlled Trials—and eight Chinese electronic bibliographic databases—China National Knowledge Infrastructure, Wan Fang Database, SinoMed, Chinese Medical Current Contents, HK Index to Chinese Periodical Literature, HyRead, Taiwan Electronic Periodical Services, and Airiti Library—were searched from January 2009 to August 2017. The initial key words were “stroke,” “acupuncture,” “needl*,” “shoulder pain,” “range of motion,” “extremity function*,” “?edema,” “physical function*,” “quality of life,” “depression,” and “self?esteem.” Variations of different terms were used to make a systematic search (Data Sheet S2 in Supplementary Material). Similar but specific search combinations of the key word variations were used for particular databases.

Two independent reviewers (Xingfeng Yu and Suzanne Hoi Shan Lo) screened the titles of all identified records for their relevance. For records whose relevance could not be determined by their titles alone, abstracts or full texts were retrieved for further assessment. The reviewers independently reviewed the abstracts and full texts of all potential records according to the pre-specified criteria. The reference lists of all relevant records, including eligible trials, review articles, and articles reporting the use of acupuncture, were reviewed to identify potentially eligible trials. Discrepancies were solved by discussion or by consultation with the third reviewer (Janita Pak Chun Chau) if consensus could not be reached.

### Risk of Bias in Individual Studies

Two independent reviewers (Xingfeng Yu and Janita Pak Chun Chau) assessed the risk of bias of all eligible trials using the Joanna Briggs Institute (JBI) Critical Appraisal Checklist for RCTs (26). The item-to-item judgments of the individual studies’ risk of bias were made referring to the “JBI explanation for the critical appraisal tool for RCTs with individual participants in parallel group” (26). Any disagreement was resolved by discussion with the third reviewer (Suzanne Hoi Shan Lo).

### Data Extraction

One reviewer (Xingfeng Yu) extracted the details (including the number and characteristics of the participants, the treatment regimen, the outcome variables and their measures, and the results) of the trials. The second reviewer (Janita Pak Chun Chau) checked the accuracy of the extracted data. Disagreements were resolved by consensus between the two reviewers; otherwise, the third reviewer (Suzanne Hoi Shan Lo) was consulted. For records that reported the same trial, data were extracted simultaneously to yield the most comprehensive information.

### Data Synthesis

The included trials were categorized and analyzed according to the types and outcomes of acupuncture. For incomplete and missing data, the investigators were contacted for further information. Data were summarized using RevMan 5.3 software (27). Continuous data were summarized using means and SDs.

The mean difference or standardized mean difference, with the 95% confidence interval, was calculated for the pooled effect size of continuous variables that were measured using the same instrument or different instruments, respectively. The data were pooled using the fixed-effects model if there were adequate trials with sufficient homogeneity; when substantial heterogeneity existed, the random-effects model was used. The risk of publication bias was assessed visually with funnel plots and statistically with Egger’s test, if appropriate (28). Narrative summaries were presented if there were high variability of the acupuncture treatment regimens among the various trials.

## Results

### Results of the Search

**Figure 1:**
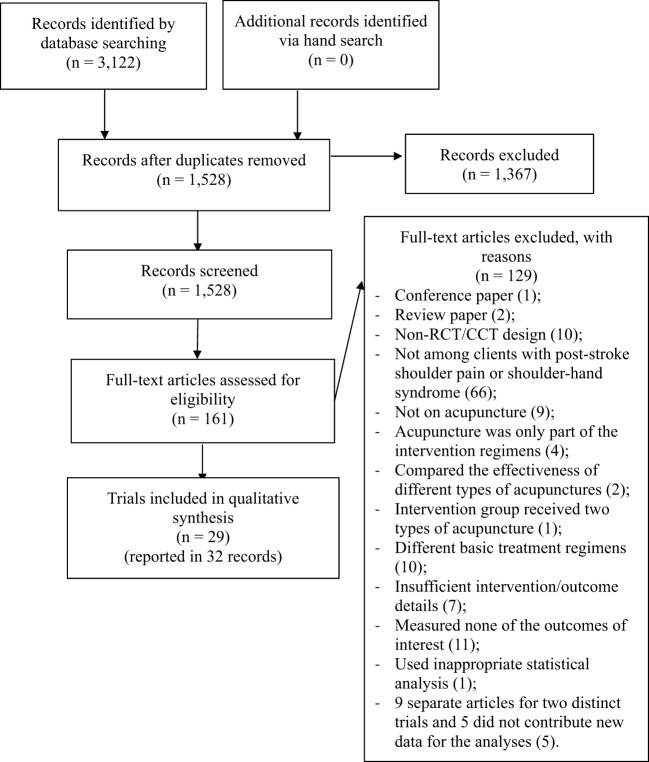
Preferred reporting items for systematic reviews and meta-analysis flow diagram of included studies.

The database search identified 3,122 records, and a manual search identified no additional trials (Figure 1). After removing duplicates, 1,528 records remained and were screened; 1,367 records were excluded due to obvious irrelevance, and the full texts of the other 161 records were retrieved for assessment. Of the records underwent full-text assessment, 13 were excluded because they were review articles or conference papers or because they used a non-RCT design. Sixty-six were excluded because the participants did not have PSP or shoulder–hand syndrome. Nine were excluded because the participants did not receive acupuncture. Two were excluded because these trials compared the effectiveness of different types of acupunctures, and another trial was excluded because the intervention group received two types of acupuncture. Four were excluded because acupuncture was only part of the intervention regimens. Ten were excluded due to differences in the basic treatment regimens. Seven were excluded due to insufficient intervention and/or outcome details despite attempts to contact the investigators. Eleven were excluded because they did not measure the outcomes of interest, and another trial was excluded due to inappropriate statistical analyses. Nine separate records for two distinct trials met the inclusion criteria, but only four contributed data for analysis. Thus, 32 records (24, 25, 29–58) reporting 29 RCTs were included in the review.

### Study Characteristics

The 29 trials were two-arm RCTs involving a total of 2,250 participants. The individual trials’ sample sizes ranged from 26 to 123 (median, 80; interquartile range, 60–90). Of the trials that provided details, the participants’ mean age ranged from 42.8 to 69.5 years; the overall percentage of male participants was 56.1%; 45.7% of the participants had stroke on the left side; and 65.2% participants had ischemic strokes. The National Institutes of Health Stroke Scale score of the participants ranged from 24.2 to 25.1, and the mean duration since the stroke ranged from 17 to 91.7 days. All participants had a clinical diagnosis of either ischemic or hemorrhagic stroke. Nine trials recruited participants with PSP, and 20 recruited those with shoulder–hand syndrome. Six trials only recruited patients with a first-ever stroke (29, 37, 40, 50, 52, 56), whereas others did not report the participants’ number of previous strokes. All trials were conducted in China. The settings included rehabilitation units of general hospitals, rehabilitation and neuromedical units, and acupuncture units in Chinese Medicine hospitals (Data Sheet S3 in Supplementary Material).

### Details of the Interventions

Of the 29 studies, 21 investigated the effects of conventional acupuncture, 5 explored the effects of electroacupuncture, 2 examined the effects of fire acupuncture, and 1 examined the effects of warm acupuncture.

#### Conventional Acupuncture

Three of the twenty-one trials on conventional acupuncture investigated the effects of traditional acupuncture combined with balance acupuncture (30, 36, 43), one investigated balance acupuncture alone (36), and the other trials explored traditional acupuncture alone.

The intervention regimens of the trials were highly heterogeneous. In total, 49 (sets of) acupoints were selected in the 21 trials, and the number of acupoints in individual trials varied from 1 to 33. All acupoints were summarized (Data Sheet S4 in Supplementary Material). The most frequently selected acupoints were Jianyu, Jianqian, Jiaoliao, Jiantong, and Jianzhen. Of the selected acupoints, Jiantong is the only one that can be used without combining with other acupoints (30, 46). The needle penetration depth of most trials was described as “deqi,” also called arrival of “qi,” which is a status that indicates appropriate acupuncture treatment (59). Among the trials that provided details, the duration of needle retention ranged from 0 to 6 h, the frequency of treatment ranged from alternate days to daily, and the number of treatment sessions ranged from 5 to 30 (Data Sheet S3 in Supplementary Material).

#### Electroacupuncture

The regimens of electroacupuncture varied considerably. The total number of treatment sessions ranged from 20 (51) to 60 (50). Four trials reported daily treatment (50, 53–55), and the other reported 1 session per day, 5 days per week (51). The number of acupoints ranged from 5 (50) to 11 (51). Jianyu, Quchi, Hegu, and Jianzhen were the most common acupoints selected. Two trials reported the depth of needle penetration: 0.8–1.2 cun under the skin (52) and “deqi” (55). Cun is a Chinese measuring unit for locating acupoints. The duration of needle retention was 30 min (52).

The electroacupuncture devices varied among the trials and included 6805-3 Electroacupuncture machine (50), SDZ-II electroacupuncture equipment (51), Han’s acupoint nerve stimulator 100A (52), 6805-A electroacupuncture device (54), and G6805 electroacupuncture machine (55). Four trials reported that the intensity of stimulation was adjusted to the level at which regular slight muscle contraction occurred and at which the client can tolerate the stimulus (51, 52, 54, 55).

#### Fire Needle Acupuncture

The acupuncture regimens of these two trials varied. The number of acupoints used was 6 (56) and 13 (57), respectively. The needle penetration under the skin was 0.2–0.5 cun for one trial (56) and 2–5 mm in the other (57); both were very shallow compared to other types of acupuncture. The duration of needle retention was 30 s in one trial (56) and was not reported in the other. Both trials adopted an alternate day treatment therapy, and the total number of sessions was 4 (56) and 11 (57), respectively.

#### Warm Needle Acupuncture

One trial investigated the effects of warm needle acupuncture (58). Seven acupoints were selected. The needle retention period for the warm needling (after the two moxa-cones placed on the acupuncture needle after insertion were completely burned out) was 30 and 30 min for traditional acupuncture. The depth of needle penetration under the skin was 5–30 mm. The treatment frequency was 1 session per day, 5 days per week, for a total of 10 sessions.

#### Usual Care

All 29 trials evaluated the effects of acupuncture treatment on the basis of different comparator interventions. Twenty-three studies used a rehabilitation program as the comparator intervention, including passive and active ROM exercise of the shoulder, positioning, alternate warm and cold bathing of the hand, ultrasonic wave treatment, and the Bobath intervention. Three studies utilized physical exercises, and three other studies prescribed herbal medicines as the comparator interventions. Twelve trials provided information related to the medical management, which included drug therapy for stroke, hypertension, and other coexisting medical conditions (24, 25, 31, 32, 34, 39, 47, 50, 52, 54, 57). No trial reported the details of the pain medication prescribed, and four trials stopped all pain medication (oral and topical) during the trial period (37, 48, 50, 52).

### Clinical Outcomes

All trials measured the outcomes immediately after completion of acupuncture treatment therapy, and one also measured the outcomes at 1-month posttreatment therapy (49). Of the 29 trials, 26 measured PSP (25, 30–43, 46–52, 54, 56–58), 6 measured edema (29, 33, 40, 54, 57, 58), 27 measured upper extremity function (25, 29–34, 36–41, 43, 45–52, 54–58), 2 measured shoulder ROM (24, 53), and 7 measured physical function (32, 37, 44, 48, 49, 52, 54). No trial measured psychosocial outcomes or reported adverse events. Shoulder pain, upper extremity function, and physical function were measured in a subjective manner using scales, whereas edema and ROM were measured with objective measurement tools, such as a ruler for ROM.

### Risk of Bias Assessment

Most studies were assessed as having a substantial risk of bias (Data Sheet S5 in Supplementary Material). Of the 29 trials, nine did not provide details on the randomization procedures (25, 31, 33, 41, 42, 44, 49, 54, 57). Allocation concealment was addressed in only one trial (48). Blinding of those who delivered acupuncture therapies was not applicable in any trial. Blinding of the outcome assessors was not reported in any trial. Only two trials reported dropouts (32, 52), but the trials did not use strategies to address the incomplete follow-up or analyze the results using the intention-to-treat principle. Sample size estimation was determined in only one trial (49). No trials reported the reliability and validity of the measurement tools, although most of these trials adopted well-established tools such as visual analog scales, the Fugl-Meyer Assessment, or the Barthel Index/Modified Barthel Index as the outcome measures.

### Effects of Acupuncture

Due to the high heterogeneity of the acupuncture therapies examined, pooling the results in a meta-analysis was not appropriate. A narrative summary of the results is thus presented.

#### Outcome: Shoulder Pain

Twenty-six trials determined the effects of acupuncture on shoulder pain among participants with PSP [19 with conventional acupuncture (25, 30–43, 46–49), 4 with electroacupuncture (50–52, 54), 2 with fire needle acupuncture (56, 57), and 1 with warm needle acupuncture (58)].

Eighteen of the nineteen studies of the effects of conventional acupuncture reported favorable effects of the treatment in reducing shoulder pain immediately after completion of the treatment regimen, whereas the other reported no significant difference between groups (34). Only one of the 18 studies measured longer term follow-up outcomes (1 month after the treatment therapy); it reported sustained favorable effects. All studies on electroacupuncture, fire needle acupuncture, and warm needle acupuncture demonstrated beneficial effects of the treatment (Data Sheet S6 in Supplementary Material).

#### Outcome: Upper Extremity Function

Twenty-seven trials determined the effects of acupuncture on upper extremity function among participants with PSP [19 on conventional acupuncture (25, 29–34, 36–41, 43, 45–49), 5 on electroacupuncture (50–52, 54, 55), 2 on fire needle acupuncture (56, 57), and 1 on warm needle acupuncture (58)].

All but one study on conventional acupuncture (34) showed that acupuncture treatment was effective in improving upper extremity function among stroke survivors (Data Sheet S7 in Supplementary Material).

#### Outcome: Edema

Six trials investigated the effects of acupuncture in reducing edema [three trials with conventional acupuncture (29, 33, 40) and one each with electroacupuncture (54), fire needle acupuncture (57), and warm needle acupuncture (58)]. All trials demonstrated a significant reduction of edema in the intervention group. All studies indicated that acupuncture treatment was effective in reducing edema (Data Sheet S8 in Supplementary Material).

#### Outcome: Shoulder ROM

Only two trials investigated the effects of either conventional acupuncture or electroacupuncture on the participants’ shoulder ROM. Both trials showed a significant improvement in ROM among the participants who received acupuncture (24, 53) (Data Sheet S9 in Supplementary Material).

#### Outcome: Physical Function

Seven trials (32, 37, 44, 48, 49, 52, 54) investigated the effects of acupuncture on the participants’ physical function; five examined the effects of conventional acupuncture (32, 37, 44, 48, 49), and the other two examined electroacupuncture (52, 54). All studies, except one study on conventional acupuncture (37), reported beneficial effects of acupuncture on improving physical function (Data Sheet S10 in Supplementary Material).

#### Adverse Events

None of the studies reported adverse events associated with the acupuncture therapy.

## Discussion

Poststroke shoulder pain is a common complication after stroke that greatly compromises survivors’ physical and psychosocial well-being. Little consensus exists on the best treatment options due to the large array of identified causes of PSP. Thus, we conducted this systematic review of RCTs to summarize the effects of different types of acupuncture on recovery outcomes in survivors with PSP.

### Effect of Acupuncture

Acupuncture is an important therapeutic technique of TCM that has been practiced for thousands of years. Acupuncture promotes recovery by inserting and operating acupuncture needles to the acupoints based on the TCM theory of Meridian and Collateral. It has been used to cure various diseases and conditions, including pain. The incidence of adverse effects of acupuncture is substantially lower than those of other medical treatments for pain reduction (60). Of the two existing reviews of acupuncture, both investigated the effects on pain reduction, and one also investigated the effects on upper extremity function (17). Although consistent with these two reviews, the findings of our systematic review must be interpreted with caution due to the high level of heterogeneity among the acupuncture therapies. In addition, most studies were assessed as having a substantial risk of bias.

Our systematic review found that conventional acupuncture can be associated with potential benefits in reducing pain and edema and in improving upper extremity function and physical function. The effects of acupuncture on improving shoulder ROM are in doubt because this outcome was only examined in two trials. Further trials are necessary before drawing conclusions regarding its effects on shoulder ROM. Although the intervention regimens of the eligible trials varied considerably, Jianyu, Jianqian, Jiaoliao, Jiantong, and Jianzhen were the most frequently selected acupoints for conventional acupuncture, and reaching the status of “deqi” was suggested.

To the best of our knowledge, this systematic review is the first to review the effects of electroacupuncture on PSP. Electroacupuncture might be effective in reducing shoulder pain and improving upper extremity function. Conclusions on the effects of electroacupuncture on edema, shoulder ROM, and physical function could not be drawn due to the limited number of eligible trials. Despite the highly heterogeneous intervention regimens of the trials on electroacupuncture, Jianyu, Quchi, Hegu, and Jianzhen were the most commonly selected acupoints, and reaching the status of “deqi” was suggested.

This systematic review also reviewed the effects of fire needle and warm needle acupuncture. However, based on the limited trials undertaken to date, there is limited evidence to support the use of either type of acupuncture in survivors with PSP.

One important finding of this review is the high degree of heterogeneity among the acupuncture therapies examined. Although the acupuncture therapy emphasized individualizing the regimen to the survivors’ conditions, examination of a standardized protocol to manage survivors with PSP in future studies would be necessary to enable better comparison of results across studies and meta-analyses to quantify its effects.

Most of the included trials were found to adhere poorly to the CONSORT Statement in their reporting. It is important for future studies to adhere to these guidelines strictly when conducting and reporting the trials to ensure the validity and robustness of the results, and hence to add more conclusive evidence in this field of practice.

Furthermore, all included trials were conducted in China and published in Chinese. There may be cultural issues when adopting and practicing acupuncture therapy in areas of other cultures. Explicit description of the regimen and conduct of the acupuncture therapy is crucial to enable its replication and hence the transferability of the results. Moreover, dissemination of the results in English would be a good way to move this field forward because it would help arouse clinicians’ and researchers’ interests in adopting and examining the effectiveness of acupuncture therapies for survivors with PSP. More importantly, we anticipate bringing this potentially promising traditional Chinese care practice into the international arena to help more survivors with PSP in the long run.

### Limitations

This review has several limitations. It considered only trials published in English and Chinese, and seven trials were excluded because they did not provide sufficient intervention and/or outcomes details. In addition, theses, conference papers, and gray literature were not included in the review process. Thus, the findings of this review are subject to publication bias. Meta-analysis and sensitivity analysis to investigate the effects of the treatment and the influence of treatment intensity on outcomes, respectively, were not feasible due to the high variability of the acupuncture treatment regimens among the various trials. Few trials provided information about the concurrent use of Western and Chinese medication that might have affected the trial outcomes. In addition, the very high potential for bias was prevalent in the included trials. These methodological flaws may have led to biased results in the included trials and thus in this review. These limitations should be taken into consideration when interpreting the findings.

### Implications for Research and Practice

Several research gaps were identified. The included trials focused only on the effects of acupuncture on the physical outcomes of survivors with PSP but neglected to examine the psychosocial benefits. Because stroke and PSP could cause many psychological problems, such as depression and low self-esteem, and seriously impede the survivors’ social lives (60), more attention must be paid to determine its effects on psychosocial well-being. In addition, the intervention and/or outcome details were not sufficiently reported in most trials, which makes it difficult for readers to judge their reliability and validity. Researchers should, therefore, report their trials according to the CONSORT statement. The conduct of acupuncture trials should also follow the International Standards for Reporting Interventions in Clinical Trials of Acupuncture (61). Explicit description of the acupuncture regimen is warranted. In addition, a more rigorous research design that ensures blinding of outcome assessors should be adopted. More detailed description of the plan of allocation concealment is needed. Further trials are needed to explore the best treatment intensity, including the frequency, needle retention duration, and total sessions for an effective acupuncture regimen. Further studies including a sham needling control might be helpful in assessing the true effects of acupuncture and the placebo effect of sham acupuncture on shoulder pain. Recruiting an adequate sample size based on a power calculation may help better demonstrate the effects of the true acupuncture therapy.

The findings of this review suggest that conventional acupuncture and electroacupuncture could be effective treatments for survivors with PSP. To maximize the treatment effects, acupuncture therapists or TCM practitioners are recommended to include the acupoints Jianyu, Jianqian, Jiaoliao, Jiantong, and Jianzhen in the treatment regimen for conventional acupuncture, and the inclusion of the Jianyu, Quchi, Hegu, Jianliao, and Jianzhen acupoints for electroacupuncture treatments. When delivering any kind of acupuncture treatment, therapists should ensure that the status of “deqi” is obtained. Examination of a standardized acupuncture protocol for PSP would also be helpful.

## Conclusion

This review suggests that conventional acupuncture and electroacupuncture could be effective treatments for survivors with PSP, with regard to reducing pain and improving upper extremity function and physical function. However, the results of this review should be interpreted with consideration of its limitations.

## Author Contributions

JC, SL, and XY cowrote and edited the manuscript. KC advised and checked the statistical analyses. JC, SL, XY, AL, JW, VL, WC, JYLC, and DT finalized the manuscript.

## Conflict of Interest Statement

The authors declare that the research was conducted in the absence of any commercial or financial relationships that could be construed as a potential conflict of interest.

## References

[1] Li Z Alexander SA. Current evidence in the management of poststroke hemiplegic shoulder pain: a review. J Neurosci Nurs (2015) 47(1):10–9. 10.1097/JNN.0000000000000109 25503543

[2] Sackley C Brittle N Patel S Ellins J Scott M Wright C The prevalence of joint contractures, pressure sores, painful shoulder, other pain, falls, and depression in the year after a severely disabling stroke. Stroke (2008) 39(12):3329–34. 10.1161/STROKEAHA.108.518563 18787199

[3] Paolucci S Iosa M Toni D Barbanti P Bovi P Cavallini A Prevalence and time course of post-stroke pain: a multicenter prospective hospital-based study. Pain Med (2016) 17(5):924–30. 10.1093/pm/pnv019 26814255

[4] Lindgren I Lexell J Jönsson AC Brogårdh C. Left-sided hemiparesis, pain frequency, and decreased passive shoulder range of abduction are predictors of long-lasting poststroke shoulder pain. PM R (2012) 4(8):561–8. 10.1016/j.pmrj.2012.04.007 22749605

[5] Roosink M Van Dongen RT Buitenweg JR Renzenbrink GJ Geurts AC IJzerman MJ. Multimodal and widespread somatosensory abnormalities in persistent shoulder pain in the first 6 months after stroke: an exploratory study. Arch Phys Med Rehabil (2012) 93(11):1968–74. 10.1016/j.apmr.2012.05.019 22683508

[6] Kalichman L Ratmansky M. Underlying pathology and associated factors of hemiplegic shoulder pain. Am J Phys Med Rehabil (2011) 90(9):768–80. 10.1097/PHM.0b013e318214e976 21430513

[7] Kim YH Jung SJ Yang EJ Paik NJ. Clinical and sonographic risk factors for hemiplegic shoulder pain: a longitudinal observational study. J Rehabil Med (2014) 46(1):81–7. 10.2340/16501977-1238 24129640

[8] Pompa A Clemenzi A Troisi E Di Mario M Tonini A Pace L Enhanced-MRI and ultrasound evaluation of painful shoulder in patients after stroke: a pilot study. Eur Neurol (2011) 66(3):175–81. 10.1159/000330657 21894021

[9] Pong YP Wang LY Huang YC Leong CP Liaw MY Chen HY. Sonography and physical findings in stroke patients with hemiplegic shoulders: a longitudinal study. J Rehabil Med (2012) 44(7):553–7. 10.2340/16501977-0987 22674236

[10] Adey-Wakeling Z Liu E Crotty M Leyden J Kleinig T Anderson CS Hemiplegic shoulder pain reduces quality of life after acute stroke: a prospective population-based study. Am J Phys Med Rehabil (2016) 95(10):758–63. 10.1097/PHM.0000000000000496 27003204

[11] Huang YC Chang KH Liou TH Cheng CW Lin LF Huang SW. Effects of Kinesio taping for stroke patients with hemiplegic shoulder pain: a double-blind, randomized, placebo-controlled study. J Rehabil Med (2017) 49(3):208–15. 10.2340/16501977-2197 28233009

[12] Kalichman L Frenkel-Toledo S Vered E Sender I Galinka T Alperovitch-Najenson D Effect of Kinesio tape application on hemiplegic shoulder pain and motor ability: a pilot study. Int J Rehabil Res (2016) 39(3):272–6. 10.1097/MRR.0000000000000167 27075946

[13] Pandian JD Kaur P Arora R Vishwambaran DK Toor G Mathangi S Shoulder taping reduces injury and pain in stroke patients: randomized controlled trial. Neurology (2013) 80(6):528–32. 10.1212/WNL.0b013e318281550e 23345636

[14] Zeferino SI Aycock DM. Poststroke shoulder pain: inevitable or preventable? Rehabil Nurs (2010) 35(4):147–51. 10.1002/j.2048-7940.2010.tb00040.x 20681389

[15] Su J Yuan S Yao C The Yellow Emperor’s Classic of Internal Medicine. Hong Kong: Chung Hwa Book Co. (HK) Ltd. (2012).

[16] Lee JA Park SW Hwang PW Lim SM Kook S Choi KI Acupuncture for shoulder pain after stroke: a systematic review. J Altern Complement Med (2012) 18(9):818–23. 10.1089/acm.2011.0457 22924414 PMC3429280

[17] Lee SH Lim SM. Acupuncture for poststroke shoulder pain: a systematic review and meta-analysis. Evid Based Complement Alternat Med (2016) 2016:3549878. 10.1155/2016/3549878 27547224 PMC4983325

[18] Moher D Liberati A Tetzlaff J Altman DG PRISMA Group Preferred reporting items for systematic reviews and meta-analyses: the PRISMA statement. Ann Intern Med (2009) 151(4):246–9. 10.7326/0003-4819-151-4-200908180-00135 19622511

[19] Sacco RL Kasner SE Broderick JP Caplan LR Connors JJ Culebras A An updated definition of stroke for the 21st century: a statement for healthcare professionals from the American Heart Association/American Stroke Association. Stroke (2013) 44(7):2064–89. 10.1161/STR.0b013e318296aeca 23652265 PMC11078537

[20] Price CI Pandyan AD. Electrical stimulation for preventing and treating post-stroke shoulder pain. Cochrane Database Syst Rev (2000) 4:CD001698. 10.1002/14651858.CD001698 11034725 PMC8406756

[21] Abdi S Complex Regional Pain Syndrome in Adults: Pathogenesis, Clinical Manifestations, and Diagnosis. (2017). Available from: https://www.uptodate.com/contents/complex-regional-pain-syndrome-in-adults-pathogenesis-clinical-manifestations-and-diagnosis

[22] Wang WY A latest theoretical study of balanced acupuncture. Chin Med Mod Distance Educ China (2004) 2:18–21. 10.3969/j.issn.1672-2779.2004.12.007

[23] Dharmananda S Electro-Acupuncture. (2002). Available from: http://www.itmonline.org/arts/electro.htm

[24] Liu JH Han SK Cao WJ Sun ZY Zuo YF Effect of Buqi Huatan Tongluo recipe combined with interior-exterior meridians acupuncture on shoulder pain of shoulder-hand syndrome after stroke. Chin Arch Tradit Chin Med (2012) 2:2674–6. 10.13193/j.archtcm.2012.12.84.liujk.070

[25] Yang WH Yang GF Han SK Effects of acupuncture combined with herbal medicine treatments for post-stroke shoulder hand syndrome. J Emerg Tradit Chin Med (2011) 20:1404, 1405–12. 10.3969/j.issn.1004-745X.2011.09.016

[26] Aromataris E Munn Z Joanna Briggs Institute Reviewers’ Manual. Australia: The Joanna Briggs Institute (2017). Available from: https://reviewersmanual.joannabriggs.org/display/MANUAL/About+this+Manual.

[27] [Computer Program]. RevMan: Review Manager (RevMan). Version 5.3. Copenhagen: The Nordic Cochrane Centre, the Cochrane Collaboration (2014).

[28] Ioannidis JP Trikalinos TA. The appropriateness of asymmetry tests for publication bias in meta-analyses: a large survey. CMAJ (2007) 176(8):1091–6. 10.1503/cmaj.060410 17420491 PMC1839799

[29] Huang CS Fan WC Yu AS Cui X Wu J Penetration acupuncture at Baxie (EX-UE 9) combined with rehabilitation for swelling hand of poststroke shoulder-hand syndrome. Chin Acupunct Moxi (2017) 37:121–4. 10.13703/j.0255-2930.2017.02.004 29231471

[30] Wu DJ Wu ZJ Liu WY Effects of acupuncture combined with rehabilitation for patients with shoulder hand syndrome after stroke. J Pract Tradit Chin Med (2017) 33:169–70. 10.3969/j.issn.1004-2814.2017.02.045

[31] Chen J Effects of acupuncture combined with exercise for patients with post-stroke shoulder pain. Womens Health Res (2016) 9:79–81.

[32] He SS Gao SY Evaluation of abdominal acupuncture and rehabilitation treatment for shoulder-hand syndrome (period 1) after stroke. J Clin Acupunct Moxi (2016) 32:11–3.

[33] Tang D Wu WP Sun XH A randomized controlled trial on the effects of meridians-based acupuncture combined with function training for shoulder hand syndrome after stroke. J Clin Acupunct Moxi (2016) 32:26–9.

[34] Wu MB Liao RX Yang HH Li N Ling HL Liu XH Observation on the clinical effects of the internal and external combined with sequential therapy for treating shoulder-hand syndrome. China Med Pharm (2016) 6:13–7.

[35] Zhou XY Chen WG Effects of intradermal needle retention combined with acupuncture for patients with post-stroke shoulder pain. Med Forum (2016) 20:4875–6. 10.3969/j.issn.1672-1721.2016.34.077

[36] Zhong CQ Ni DL Lin WJ Chen FH Effects of acupuncture combined with rehabilitation for patients with hand shoulder syndrome after stroke. Hainan Med J (2016) 27:1687–8. 10.3969/j.issn.1003-6350.2016.10.048

[37] Chen Y Huang TS Liu KC Clinical research of using acupuncture and rehabilitation training in the treatment of post-stroke shoulder-hand syndrome stage I. J Sichuan Tradit Chin Med (2015) 33:150–2.

[38] Li B Treating 57 cases of stroke shoulder-hand syndrome by acupuncture. Clin J Chin Med (2015) 7:40–1. 10.3969/j.issn.1674-7860.2015.19.020

[39] Wu JY Ye BY Xue XH Huang SE Lin ZC Hong JC Observations on the efficacy of wrist-ankle acupuncture plus continuous exercise therapy for post-stroke shoulder pain. Shang J Acupunct Moxi (2015) 34:409–11. 10.13460/j.issn.1005-0957.2015.05.0409

[40] Xu F Li HL Zhang Z A randomized controlled trial on the effectiveness of acupuncture combined with rehabilitation for post-stroke shoulder hand syndrome. Chin J Trauma Disabil Med (2015) 23:141–2. 10.13214/j.cnki.cjotadm.2015.16.107

[41] Zhang XR Lu WX The effects of acupuncture combined with rehabilitation for stage I shoulder hand syndrome patients. China Med Eng (2015) 23:200.

[42] Zhang ZY Zhang MB A randomized controlled trial on the effects of acupuncture combined with traditional Chinese medicine syndrome differentiation treatment on hemiplegic shoulder pain. J Pract Tradit Chin Intern Med (2015) 29:23–5. 10.13729/j.issn.1671-7813.2015.11.11

[43] Lin HX Ye GQ Liao HX Lin FY Liang BJ Acupuncture combined with rehabilitation training in the treatment of shoulder-hand syndrome after stroke. World Chin Med (2014) 9:84, 85–8. 10.3969/j.issn.1673-7202.2014.01.030

[44] Sun ZY Han SK Cao WJ Liu JH Zuo LQ Lin GQ Effects of Buqi Huatan Tongluo recipe combined with interior-exterior meridians acupuncture on spasticity relief for patients with shoulder hand syndrome after stroke. Shaanxi J Tradit Chin Med (2013) 34:1004–6. 10.3969/j.issn.1000-7369.2013.08.039

[45] Han SK Effect of Buqi Huatan Tongluo recipe combined with interior-exterior meridians acupuncture for patients with shoulder-hand syndrome after stroke. J New Chin Med (2011) 43:97–9. 10.13457/j.cnki.jncm.2011.01.024

[46] Sun YZ Wang YJ Wang W Effect of acupuncture plus rehabilitation training on shoulder-hand syndrome due to ischemic stroke. J Acupunct Tuina Sci (2012) 10:109–13. 10.1007/s11726-012-0583-z

[47] Zhang ZX Zhang Y Yu TY Gao HY The effects of acupuncture on Jiantong point combined with exercise for post-stroke shoulder pain patients. Shandong Med J (2012) 52:82–3. 10.3969/j.issn.1002-266X.2012.27.032

[48] Chen HX He MF Xie RM Clinical observation on the combination of abdominal acupuncture and rehabilitation in treating omalgia after stroke. J Nanjing Univ Tradit Chin Med (2011) 27:333–5. 10.14148/j.issn.1672-0482.2011.04.006

[49] Shi DK Tang XS Carpus-ankle acupuncture combined with physical therapy for patients with post-stroke shoulder pain: a randomized controlled trial. J Chengdu Univ Tradit Chin Med (2011) 34:33–5. 10.13593/j.cnki.51-1501/r.2011.01.012

[50] Bu L Xu HQ Tan WJ Di RK Effects of electro-acupuncture combined with scapular control training on shoulder pain and upper limbs function in hemiplegia patients. Glob Tradit Chin Med (2013) 6:246–7.

[51] Jia CJ Ni GX Tan H Zhang X Effects of acupuncture combined with rehabilitation for stroke survivors with stage I shoulder hand syndrome. J Changchun Univ Tradit Chin Med (2012) 4:711–2. 10.13463/j.cnki.cczyy.2012.04.084

[52] Bao YH Wang YW Chu JM Zhu GX Wang CM Hou HM Effects of electro-acupuncture combined with rehabilitation for patients with post-stroke shoulder pain. Chin Arch Tradit Chin Med (2011) 29:2536–9. 10.13193/j.archtcm.2011.11.162.baoyh.038

[53] Bao YH Wang YW Chu JM Zhu GX Wang CM Hou HM Effects of electro-acupuncture combined with rehabilitation on improving upper extremity function for patients with post-stroke shoulder pain. Chin J Tradit Med Sci Tech (2012) 19:59–60. 10.3969/j.issn.1005-7072.2012.01.041

[54] Hong LR Chen B Yu SM Huang XS Wang JP Xia Y Efficacy of acupuncture plus rehabilitation training in treating shoulder-hand syndrome after hemiparalysis. Med J Chin Peoples Armed Police Forces (2011) 22:658–60. 10.3969/j.issn.1004-3594.2011.08.005

[55] Yang D Xie M Zhang CE Ye BY Song GM Effects of electro-acupuncture combined with rehabilitation for patients with shoulder hand syndrome. Liaoning J Tradit Chin Med (2009) 36:1770–1. 10.13192/j.ljtcm.2009.10.142.yangd.082

[56] Xu ZQ Wang SX Zhou ZH Clinical studies on fire needle treatment of stroke shoulder-hand syndrome stage I. Chin J Chin Med (2016) 31:753–5, 760. 10.16368/j.issn.1674-8999.2016.05.211

[57] Wang X Wang WQ Clinical studies on the effectiveness of fire needle treatment for patients with shoulder hand syndrome. Guangming J Chin Med (2011) 26:754–6. 10.3969/j.issn.1003-8914.2011.04.079

[58] Nie WB Zhao H Clinical study on the treatment of shoulder-hand syndrome by warming and dredging triple energizer plus rehabilitation training. Shanghai J Acupunct Moxi (2011) 30:217–9. 10.3969/j.issn.1005-0957.2011.04.217

[59] Yang XY Shi GX Li QQ Zhang ZH Xu Q Liu CZ. Characterization of Deqi sensation and acupuncture effect. Evid Based Complement Alternat Med (2013) 2013:319734. 10.1155/2013/319734 23864884 PMC3705793

[60] National Institutes of Health Consensus Conference. Acupuncture. JAMA (1998) 280(17):1518–24. 10.1001/jama.280.17.1518 9809733

[61] MacPherson H Altman DG Hammerschlag R Youping L Taixiang W White A Revised Standards for Reporting Interventions in Clinical Trials of Acupuncture (STRICTA): extending the CONSORT statement. J Evid Based Med (2010) 3(3):140–55. 10.1111/j.1756-5391.2010.01086.x 21349059

