# Supplementary material for: Effects of Acupuncture on the Recovery Outcomes of Stroke Survivors with Shoulder Pain: A Systematic Review
Source: Front Neurol. 2018 Jan 31;9:30. doi: 10.3389/fneur.2018.00030 (PMC5797784; doi:10.3389/fneur.2018.00030)
Supplement: Supplementary file 2 [file Data_Sheet_2.DOCX]

**Supplementary Data 2: An example of search strategy**

**MEDLINE**

1. exp Stroke/ or exp Cerebrovascular Disorders/ or exp Intracranial Hemorrhages/ or exp Cerebral Hemorrhage/ or exp Cerebral Infarction/
2. (stroke* or cva* or apoplex* or cerebral vascular* or post stroke* or poststroke* or SAH or ICH).tw.
3. ((cerebrovascular* or cerebro vascular* or brain vascular*) adj3 (disease* or accident* or disorder* or attack* or event*)).tw.
4. ((cerebral* or brain* or cerebell* or vertebrobasilar* or intracerebral* or intra cerebral* or intracran* or intra cran*) adj3 (isch?em* or infarct* or thrombo* or embol* or occlus*)).tw.
5. ((cerebral* or brain* or cerebell*or intracerebral* or intra cerebral* or intracran* or intra cran* or subarachnoid*) adj3 (h?emorrhag* or bleed* or h?ematoma* or infarct*)).tw.
6. or/1-5
7. exp Shoulder Pain/
8. exp Reflex Sympathetic Dystrophy/ or exp Bursitis/
9. ((shoulder* or h?emiplegic*) adj3 pain*).mp.
10. adhesive capsulitis.mp.
11. shoulder?hand syndrome.mp.
12. complex regional pain syndrome.mp.
13. (HSP or PSP or PSSP or SHS or CRPS or RSD).mp.
14. or/7-13
15. 6 and 14
16. exp Acupuncture/ or exp Acupuncture Therapy/ or exp Acupuncture Analgesia/ or exp Electroacupuncture/or exp Needles/
17. exp Electric Stimulation Therapy/ or exp Electric Stimulation/
18. (acupunct* or electro?acupuncture* or needl*).mp.
19. (electric* adj3 stimulat*).mp.
20. or/16-19
21. 15 and 20
22. limit 21 to yr="2009 -Current"

**Chinese database search**

(肩痛 OR 肩手综合征 OR 半身不遂) AND (针灸 OR 针刺 OR 肩三针 OR 肩针 OR 头针 OR 体针 OR 腹针 OR 腕踝针 OR 电针OR 温针 OR火針OR 平衡针 OR 浮刺 OR 巨刺 OR 经筋排刺)
