# Supplementary material for: Effects of Acupuncture on the Recovery Outcomes of Stroke Survivors with Shoulder Pain: A Systematic Review
Source: Front Neurol. 2018 Jan 31;9:30. doi: 10.3389/fneur.2018.00030 (PMC5797784; doi:10.3389/fneur.2018.00030)
Supplement: Supplementary file 3 [file Data_Sheet_3.DOCX]

**Supplementary Data 3: Characteristics of the included studies**

| **Authors**  **Year**  **Region**  **Sample size (IG:CG)**  **Settings** | **Participants** | | | | **Interventions** | | | | | | | **Outcomes**  **(measures)** |
| --- | --- | --- | --- | --- | --- | --- | --- | --- | --- | --- | --- | --- |
|  | **Study participants** | Gender ratio (M:F)  Stroke type (I:H)  Stroke side (L:R)  NIHSS (IG vs. CG)  Drop-out rate | **Age**  **(years, mean**±**SD)** | **Post-stroke**  **duration**  **(days, mean**±**SD)** | **Interventions** | **Acupuncture technique;**  **Needle information** | **Number of acupoints** | **Penetration**  **depth** | **Duration of needle retention** | **Frequency** | **Number of total sessions** |  |
| Huang et al.  2017  China  60 (30:30)  Reh. unit; TCM hospital | With Stage I SHS | 43:17  41:19  No info.  No info.  No info. | IG:  59±6  CG:  59±5 | IG:  46.8±14.8  CG:  45.4±16.5 | Acupuncture + rehabilitation  vs.  Rehabilitation | Even reinforcing-reducing method;  Diameter:  0.35 mm,  Length:  40 mm | Five acupoints:  Baxie (4), and Wailaogong on the hemiplegic side | 35~38 mm  (Deqi) | 20 min  (rotate the needles every 10 min) | One session per day, five days per week | 15  (3 weeks) | 1. Edema;  2. Upper extremity function  (FMA) |
| Wu et al.  2017  China  100 (50:50)  Reh. unit; general hospital | With Stage I SHS | 57:43  No info.  No info.  24.3±5.3 vs. 24.6±5.2  No info. | IG:  60.3±5.3  CG:  60.5±5.6 | IG:  28.6±5.8  CG:  29.2±5.6 | Acupuncture + rehabilitation  vs.  Rehabilitation | Clockwise insertion;  No needle information. | Three + Seven acupoints:  Balance acupuncture: Shengti, Jiantong, and Piantan on the non-hemiplegic side;  Traditional acupuncture: Ashi, Neiguan, Jiansanzhen (3), Chize and Jiquan | Deqi | Balance acupuncture: none;  Ordinary acupuncture: 30min | Daily | 30  (30 days) | 1. Pain  (VAS);  2. Upper extremity function  (FMA) |
| Chen  2016  China  80 (40:40)  Neuromedical unit;  Reh. hospital | With PSP | 43:37  Unclear info.  Unclear info.  No info.  No info. | 62.4±2.2 | 44.3±2.4 | Acupuncture + physical exercise  vs.  physical exercise | Twirling lifting and thrusting insertion;  No needle information | One acupoint:  Jiantong | 2 Cun | 10~20 min | Daily | 21  (3 weeks) | 1. Pain  (VAS);  2. Upper extremity function  (FMA) |
| He & Gao  2016  China  123 (62:61)  Reh. unit;  TCM hospital | With Stage I SHS | Unclear info.  Unclear info.  No info.  No info.  IG: 3.2%; CG: 1.6% | IG:  50.7±10.2  CG:  48.1±12.0 | IG:  21.1±8.7  CG:  20.3±8.6 | Acupuncture + rehabilitation  vs.  rehabilitation | Perpendicular insertion;  No needle information | Eight acupoints:  Zhongwan, Guanyuan, Huaroumen on both sides, Wailing on both sides, Shangfengshidian on the hemiplegic side, Shangfengshiwaidian on the hemiplegic side | Zhongwan and Guanyuan: muscle;  Wailing on both sides and Huaroumen on the non-hemiplegic side: superficial fascia;  Huaroumen on the hemiplegic side, shangfengshidian and shangfengshiwaidian: epidermis | 30 min | Daily | 21  (3 weeks) | 1. Pain  (VAS);  2. Upper extremity function  (FMA);  3. Physical function (MBI) |
| Tang et al.  2016  China  60 (30:30)  Acup. unit and Reh. unit;  TCM hospitals | With Stage I SHS | 39:21  No info.  No info.  No info.  No info. | IG:  57.9±8.2  CG:  59.3±8.5 | IG:  48.5±9.6  CG:  49.1±9.4 | Acupuncture + functional exercise  vs.  functional exercise | No detail on acupuncture technique;  Diameter:  0.35 mm,  Length:  40 mm | Six acupoints:  Tianzong, Jianyu Binao, Jianqian, Zhongzhu and Houxi on the hemiplegic side | Deqi | 40 min | One session per day, six days a week | 24  (4 weeks) | 1. Pain  (VAS);  2. Edema;  3. Upper extremity function  (FMA) |
| Wu et al.  2016  China  120 (60:60)  Integrative medicine unit, general hospital | With Stage I SHS | 49:71  No info.  No info.  No info.  No info. | IG:  42.4±3.1  CG:  43.1±2.0 | No info. | Acupuncture + rehabilitation  vs.  rehabilitation | Perpendicular insertion;  No needle information | Eight acupoints:  Five-shu points (Jing-well, Xing-spring, Shu-stream, Jing-river, He-sea), Jianjing, Jianliao, Jianzhen | Deqi | 30 min | Daily | 21  (3 weeks) | 1. Pain  (VAS);  2. Upper extremity function  (FMA) |
| Zhou & Chen  2016  China  26 (14:12)  General hospital | With PSP | 15:11  18:8  No info.  No info.  No info. | IG:  56.6±10.7  CG：  56.3±11.2 | No info. | Acupuncture + rehabilitation  vs.  rehabilitation | No detail on acupuncture technique;  No needle information | Eight to nine acupoints:  Dingpangxian II (MS9), Jianyu, Jianliao, Jianzhen, Jianqian, Tianzong, Quchi, Waiguan, Ashi (depends) | No info. | 30 min | Daily | 28  (4 weeks) | 1. Pain  (VAS) |
| Zhong et al.  2016  China  60 (30:30)  Reh. unit; general hospital | With Stage I SHS | 35:25  No info.  No info.  24.8±5.4 vs. 24.7±5.1  No info. | IG:  62.5±7.4  CG:  62.3±8.9 | IG:  28.4±10.3  CG:  29.2±9.8 | Acupuncture + rehabilitation  vs.  rehabilitation | Clockwise insertion;  No needle information | Three + Seven acupoints:  Balance acupuncture: Piantan, Jiantong, and Shengti on the non-hemiplegic side;  Traditional acupuncture: Jiansanzhen (3), Ashi, Jiquan, Neiguan and Chize | Deqi | Balance acupuncture: none;  Ordinary acupuncture: 30min | Daily | 30  (30 days) | 1. Pain  (VAS);  2. Upper extremity function  (FMA) |
| Chen et al.  2015  China  94 (48:46)  Reh. unit; general hospital | With Stage I SHS | 43:51  54:40  No info.  No info.  No info. | IG:  58.3±11.6  CG:  60.1±12.5 | IG:  34.9±14.7  CG:  35.4±16.9 | Acupuncture + rehabilitation  vs.  rehabilitation | Twirling lifting and thrusting insertion;  Diameter:  0.3 mm,  Length:  40 mm | Eleven acupoints:  Jianyu, Jianliao, Jianqian, Jianzhen, Quchi, Waiguan, Shousanli and Baxie (4) on the hemiplegic side | Deqi | 30 min  (rotate the needles every 10 min) | One session per day, six days a week | 24  (4 weeks) | 1. Pain  (VAS);  2. Upper extremity function  (FMA);  3. Physical function (BI) |
| Li  2015  China  114 (57:57)  Reh. unit; general hospital | With Stage I SHS | 75:39  No info.  No info.  25.1±1.9 vs. 25.4±2.1  No info. | IG:  64.3±5.9  CG:  63.5±6.4 | IG:  33.5±7.9  CG:  31.6±7.4 | Acupuncture + rehabilitation  vs.  rehabilitation | Twirling lifting and thrusting insertion;  Diameter:  0.3 mm,  Length:  75 mm | Four acupoints:  Shengti, Piantan, Jiantong, Xitong | Deqi | None | Daily | 28  (4 weeks) | 1. Pain  (VAS);  2. Upper extremity function  (FMA) |
| Wu et al.  2015  China  80 (40:40)  Reh. unit;  TCM hospital | With PSP | 41:39  50:30  No info.  No info.  No info. | IG:  69±8  CG:  70±8 | IG:  56.2±17.3  CG:  52.4±15.2 | Acupuncture + rehabilitation  vs.  rehabilitation | Puncturing through the skin tissue and then insert in the direction parallel with the skin;  No needle information | Two acupoints:  Wrist 4 and Wrist 5 on the hemiplegic side | 35 mm | 6 h | One session per day, six days a week | 24  (4 weeks) | 1. Pain  (VAS);  2. Upper extremity function  (FMA) |
| Xu et al.  2015  China  80 (40:40)  Reh. unit;  TCM hospital | With Stage I SHS | 49:31  80:0  No info.  No info.  No info. | IG:  59.6±8.7  CG:  60.3±9.1 | IG:  47.2±9.6  CG:  48.6±9.3 | Acupuncture + rehabilitation  vs.  rehabilitation | Twirling lifting and thrusting insertion;  No needle information | Seven acupoints:  Jianyu, Binao, Jiquan, Jianqian, Jianhou, Zhongzhu and Houxi on the hemiplegic side | Deqi | 40 min  (rotate the needles every 10 min) | Daily | 14  (14 days) | 1. Pain  (VAS);  2. Edema;  3. Upper extremity function  (FMA) |
| Zhang & Lu  2015  China  92 (46:46)  General hospital | With I SHS | 53:39  No info.  No info.  No info.  No info. | IG:  60.2±4.9  CG:  61.3±4.7 | IG:  29.6±5.5  CG:  30.1±6.1 | Acupuncture + rehabilitation  vs.  rehabilitation | No detail on acupuncture technique;  Diameter:  0.3 mm,  Length:  40 mm | Six acupoints:  Baihui, Shenting, Yintang, Quchi, Waiguan, Hegu | No info. | 30 min | One session per day, five days a week | 20  (4 weeks) | 1. Pain  (VAS);  2. Upper extremity function  (FMA) |
| Zhang & Zhang  2015  China  53 (27:26)  Acup. clinic;  TCM hospital | With PSP | 22:31  No info.  No info.  No info.  No info. | IG:  59.3±9.4  CG:  61.9±8.6 | IG:  87.7±31.2  CG:  95.9±34.9 | Acupuncture + herbal medicine treatment  vs.  herbal medicine treatment | Even reinforcing-reducing method;  No needle information | Eight to nine acupoints:  Shuigou, Neiguan, Sanyinjiao, Jiquan, Tongli, Zusanli, and Yongquan;  plus  Jianqian and Lieque, or, Jianzhen and Houxi, or Waiguan, or, Quchi and Jianliao, or Guanyuan | Deqi | 30 min except Jiquan (no needle retention)  (rotate the needles every 10 min) | Daily | 28  (4 weeks) | 1. Pain  (VAS) |
| Lin et al.  2014  China  80 (40:40)  Reh. unit; general hospital | With Stage I SHS | 43:37  No info.  No info.  24.4±4.5 vs. 24.2±4.2  No info. | IG:  62.6±8.9  CG:  62.1±8.6 | IG:  29.3±8.7  CG:  28.6±8.1 | Acupuncture + rehabilitation  vs.  rehabilitation | Clockwise insertion;  No needle information | Three + Seven acupoints:  Balance acupuncture: Piantan, Jiantong, and Shengti on the non-hemiplegic side;  Traditional acupuncture: Jiansanzhen (3), Ashi, Jiquan, Neiguan and Chize on the hemiplegic side | Deqi | Balance acupuncture: none;  Ordinary acupuncture: 30min  (rotate the needles every 15 min) | Daily | 28  (4 weeks) | 1. Pain  (VAS);  2. Upper extremity function  (FMA) |
| Han et al.  2013, 2012, 2011,  China  90 (50:40)  Neuromedical unit; a TCM hospital and a general hospital | With Stage I SHS | 53:37  47:43  No info.  No info.  No info. | IG:  54.7±2.9  CG:  55.7±2.2 | IG:  25.2±7.1  CG:  26.3±8.8 | Acupuncture + herbal medicine treatment  vs.  herbal medicine treatment | Bird-pecking twirling insertion;  Diameter:  0.3 mm,  Length:  46 mm | Thirty-three acupoints:  Five-shu points (Jing-well, Xing-spring, Shu-stream, Jing-river, He-sea) of the Taiyin Lung Meridian of Hand, Yangming Large Intestine Meridian of Hand, Jueyin Pericardium Meridian of Hand, Shaoyang Sanjiao Meridian of Hand, Shaoyin Heart Meridian of Hand, and Taiyang Small Intestine Meridian of Hand, Jianjing, Jianliao, and Jianzhen | No info. | 30 min | Daily | 21  (3 weeks) | 1. Upper extremity function  (FMA);  2. Physical function (MBI);  3. Shoulder range of motion |
| Yang et al.  2011  China  100 (50:50)  Emergency unit, Reh. Unit, and integrative medicine unit; general hospital | With Stage I SHS | 53:47  No info.  No info.  No info.  No info. | IG:  54.7±2.9  CG:  55.7±2.2 | IG:  25.2±7.1  CG:  26.3±8.8 | Acupuncture + herbal medicine treatment  vs.  herbal medicine treatment | Bird-pecking twirling insertion;  Diameter:  0.3 mm,  Length:  46 mm | Thirty-three acupoints:  Five-shu points (Jing-well, Xing-spring, Shu-stream, Jing-river, He-sea) of the Taiyin Lung Meridian of Hand, Yangming Large Intestine Meridian of Hand, Jueyin Pericardium Meridian of Hand, Shaoyang Sanjiao Meridian of Hand, Shaoyin Heart Meridian of Hand, and Taiyang Small Intestine Meridian of Hand, Jianjing, Jianliao, and Jianzhen | No info. | 30 min | Daily | 28  (4 weeks) | 1. Pain  (VAS);  2. Upper extremity function  (FMA) |
| Sun et al.  2012  China  60 (30:30)  Acup. unit;  TCM hospital | With Stage I SHS | 39:21  No info.  No info.  No info.  No info. | IG:  60.4±8.7  CG:  59.2±8.5 | IG:  45.2±9.6  CG:  46.7±9.1 | Acupuncture + rehabilitation  vs.  rehabilitation | Mild twirling insertion;  Diameter:  0.35 mm,  Length:  40 mm | Two to eight acupoints:  Jianqian and Yuji / Jianyu and Hegu / Jianliao and Zhongzhu / Naoshu and Houxi | Deqi | 20 min | One session per day, six days one week | 18  (3 week) | 1. Pain  (VAS);  2. Upper extremity function  (FMA) |
| Zhang et al.  2012  China  80 (40:40)  Reh. unit; general hospital | With PSP | 45:35  26:54  35:45  No info.  No info. | 56±16.7 | 10~102 | Acupuncture + physical exercise  vs.  physical exercise | Twirling lifting and thrusting insertion;  Diameter:  0.25 mm,  Length:  40 mm | One acupoint:  Jiantong | 2 Cun  (Deqi) | 10~20 min | Daily | 21  (3 weeks) | 1. Pain  (VAS);  2. Upper extremity function  (FMA) |
| Chen et al.  2011  China  60 (30:30)  Reh. unit;  TCM hospital | With PSP | 35:25  No info.  No info.  No info.  No info. | IG:  64.1±13.3  CG:  62.8±10.5 | IG:  40.2±10.9  CG:  41.6±9.8 | Acupuncture + rehabilitation  vs.  rehabilitation | Perpendicular insertion;  Diameter:  0.22 mm,  Length:  25 mm | Five acupoints:  Zhongwan, Shangqu, Wailing, Shangfengshidian, Shangfengshiwaidian | No info. | 30 min | One session per day, six days one week | 12  (2 weeks) | 1. Pain  (VAS);  2. Upper extremity function  (FMA);  3. Physical function (MBI) |
| Shi & Tang  2011  China  90 (45:45)  Integrative hospital | With PSP | No info.  No info.  No info.  No info.  No info. | No info. | No info. | Acupuncture + rehabilitation  vs.  rehabilitation | Puncturing through the skin tissue and then insert in the direction parallel with the skin;  No needle information | Three acupoints:  Upper 4, 5 and 6 of the upper extremity on the hemiplegic side | 35 mm  (Deqi) | 30 min | Every two days | 5  (10 days) | 1. Pain  (VAS);  2. Upper extremity function  (FMA);  3. Physical function (BI) |
| Bo et al.  2013  China  67 (34:33)  Reh. unit; general hospital | PSSP patients | 34:33  45:22  No info.  No info.  No info. | IG:  64.7±9.7  CG:  65.1±9.2 | IG:  78.6±43.8  CG:  77.1±42.3 | Electro-acupuncture + rehabilitation  vs.  rehabilitation | Introduce electricity stimulation to the needles on Jianyu and Jianliao, or Jianzhen and Jianqian;  No needle info. | 5  Jianyu, Jianzhen, Biliao, Jianqian, and Quchi | No info. | 20 min | Daily | 60  (2 months) | 1. Pain  (VAS);  2. Upper extremity movement function  (FMA) |
| Jia et al.  2012  China  52 (28:24)  Reh. unit and neuromedical unit; general hospital | Stage I SHS patients | 31:21  No info.  No info.  No info.  No info. | IG:  60.5±5.3  CG;  61.3±5.9 | IG:  28.5±5.7  CG:  31.0±7.3 | Electro-acupuncture + rehabilitation  vs.  rehabilitation | Insert into the acupoints using even reinforcing-reducing method, then introduce electrical stimulation to the needles;  0.3*40 mm | 8~11  Baihui, Shenting, Yintang, Jianyu, Jianliao, Quchi, Waiguan, Hegu;  For patients with serious upper extremity spasm, add Jiquan, Chize and Neiguan;  For patients with serious pain, add Ashi | No info. | 30 min | One session per day, five days one week | 20  (4 weeks) | 1. Pain  (VAS);  2. Upper extremity movement function  (FMA) |
| Bao et al.  2012, 2011  China  88  (46: 42)  Acup. unit;  TCM hospital | PSSP patients | 45:43  65:23  No info.  No info.  IG: 0%; CG: 10.6% | IG:  67.4±9.8  CG:  64.9±8.9 | IG:  78.9±42.6  CG:  77.7±42.3 | Electro-acupuncture + rehabilitation  vs.  rehabilitation | Insert into the acupoints, then introduce electrical stimulation to the needles;  0.25*40 mm | 7  Jianyu, Jianliao, Jianzhen, Binao, Quchi, Shousanli, and Waiguan | 0.8~1.2 Cun | 30 min | Daily | 30  (1 month) | 1. Pain  (NPRS);  2. Upper extremity movement function  (FMA);  3. Activity function (BI);  4. Shoulder range of motion |
| Hong et al.  2011  China  82 (41:41)  Reh. hospital | Stage I SHS patients | 44:38  Unclear info.  39:43  No info.  No info. | IG:  48.2±6.0  CG:  50.0±6.0 | IG:  30.8±3.6  CG:  31.4±4.1 | Electro-acupuncture + rehabilitation  vs.  rehabilitation | Insert into the acupoints, then introduce electrical stimulation to the needles;  No needle info. | 8  Hegu, Shousanli, Quchi, Jianyu, Jianzhen, Tianzong, Waiguan and Jianliao on the hemiplegic side | No info. | 20 min | Daily | 42  (6 weeks) | 1. Pain  (VAS);  2. Edema;  3. Upper extremity movement function  (FMA)  4. Activity function (BI) |
| Yang et al.  2009  China  50 (25:25)  Acup. unit; TCM hospital | Stage I SHS patients | 28:22  33:17  No info.  No info.  No info. | IG:  58.1±8.2  CG:  56.1±8.4 | IG:  45±15  CG:  46±14 | Electro-acupuncture + rehabilitation  vs.  rehabilitation | Insert into the acupoints, then introduce electrical stimulation to the needles;  No needle info. | 6  Jianjing, Jianyu, Jianzhen, Binao, Shousanli, and Hegu | Deqi | 30 min | Daily | 30  (1 month) | 1. Upper extremity movement function  (FMA) |
| Xu et al.  2016  China  84 (42:42)  TCM hospital | Stage I SHS patients | 45:39  49:35  No info.  No info.  No info. | IG:  51.1±12.3  CG:  54.2±14.3 | IG:  32.5±13.5  CG:  34.9±14.2 | Fire acupuncture + rehabilitation  vs.  rehabilitation | Heat the needles on fire and then insert perpendicularly into the acupoints;  0.34*25 mm | 6  Jianyu, Jianliao, Ashi, Quchi, Waiguan, Hegu | 0.2~0.5 Cun | 30 s | Every two days | 4  (8 days) | 1. Pain  (VAS);  2. Upper extremity movement function  (FMA) |
| Wang & Wang  2011  China  85 (43:42)  Acup. clinic and unit; general hospital | Stage I SHS patients | 56:29  68:17  No info.  No info.  No info. | IG:  65.7±10.3  CG:  64.3±9.6 | IG:  72.9±34.3  CG:  70.3±35.2 | Fire acupuncture + rehabilitation  vs.  rehabilitation | Heat the needles on fire and then insert into the acupoints;  No needle info. | 13  Jianqian, Jianyu, Jianzhen, Quchi, Waiguan, Baxie (4), Shuifen, and Zhongwan on the hemiplegic side; Yinlingquan and Fenglong on the non-hemiplegic side | 2~5 mm | No info. | Every two days | 11  (3 weeks) | 1. Pain  (VAS);  2. Edema;  3. Upper extremity movement function  (FMA) |
| Nie & Zhao  2011  China  40 (20:20)  Acup. clinic and unit; TCM hospital | Stage I SHS patients | 27:13  No info.  No info.  No info.  No info. | IG:  68±6  CG:  67±6 | IG:  18.1±4.8  CG:  16.8±4.2 | Warm acupuncture + rehabilitation  vs.  rehabilitation | Insert perpendicularly into the acupoints using even reinforcing-reducing method, and then fire the moxa on the end of the needles on Waiguan and Yangchi;  0.3*25 mm or 0.3*50 mm | 7  Waiguan, Yangchi, Wangu, Hegu, Quchi, Jianyu, and Jianjing on the hemiplegic side | 5~30 mm | Warm acupuncture:  time for 2 moxa-cones to burn out;  ordinary acupuncture:  30 min | One session per day, five days one week | 10  (2 weeks) | 1. Pain  (VAS);  2. Edema;  3. Upper extremity movement function  (FMA) |

Note: Acup. = Acupuncture; BI = Barthel Index; CG = Control Group; Cun = a Chinese measuring unit for locating acupoints; F = Female; FMA = Fugl-Meyer Assessment; H = Hemorrhagic; I = Ischemic; IG = Intervention Group; info. = information; L = Left; M = Male; MBI = Modified Barthel Index; NIHSS = National Institutes of Health Stroke Scale; NRS = Numeric Pain Rating Scale; PSP = Post-stroke shoulder pain; R = Right; Reh. = Rehabilitation; SHS = Shoulder Hand Syndrome; TCM = Traditional Chinese Medicine; VAS = Visual Analogue Scale
