# Supplementary material for: Effects of Acupuncture on the Recovery Outcomes of Stroke Survivors with Shoulder Pain: A Systematic Review
Source: Front Neurol. 2018 Jan 31;9:30. doi: 10.3389/fneur.2018.00030 (PMC5797784; doi:10.3389/fneur.2018.00030)
Supplement: Supplementary file 4 [file Data_Sheet_4.DOCX]

**Supplementary Data 4: Summary of acupoints used in the included studies**

| Acupoints | Huang et al., 2017 | Wu et al., 2017 | Chen, 2016 | He & Gao, 2016 | Tang et al., 2016 | Wu et al., 2016 | Zhou & Chen, 2016 | Zhong et al., 2016 | Chen et al., 2015 | Li, 2015 | Wu et al., 2015 | Xu et al., 2015 | Zhang & Lu, 2015 | Zhang & Zhang, 2015 | Lin et al., 2014 | Han et al., 2013, 2012, 2011 | Yang et al., 2011 | Sun et al., 2012 | Zhang et al., 2012 | Chen et al., 2011 | Shi & Tang, 2011 | Bo et al., 2013 | Jia et al., 2012 | Bao et al., 2012, 2011 | Hong et al., 2011 | Yang et al., 2009 | Xu et al., 2016 | Wang & Wang, 2011 | Nie & Zhao, 2011 |
| --- | --- | --- | --- | --- | --- | --- | --- | --- | --- | --- | --- | --- | --- | --- | --- | --- | --- | --- | --- | --- | --- | --- | --- | --- | --- | --- | --- | --- | --- |
| Baxie (EX-UE9) | √ |  |  |  |  |  |  |  | √ |  |  |  |  |  |  |  |  |  |  |  |  |  |  |  |  |  |  | √ |  |
| Wailaogong (EX-UE8) | √ |  |  |  |  |  |  |  |  |  |  |  |  |  |  |  |  |  |  |  |  |  |  |  |  |  |  |  |  |
| Shengti |  | √ |  |  |  |  |  | √ |  | √ |  |  |  |  | √ |  |  |  |  |  |  |  |  |  |  |  |  |  |  |
| Jiantong |  | √ | √ |  |  |  |  | √ |  | √ |  |  |  |  | √ |  |  |  | √ |  |  |  |  |  |  |  |  |  |  |
| Piantan |  | √ |  |  |  |  |  | √ |  | √ |  |  |  |  | √ |  |  |  |  |  |  |  |  |  |  |  |  |  |  |
| Ashi point |  | √ |  |  |  |  | √ | √ |  |  |  |  |  |  | √ |  |  |  |  |  |  | √ |  |  |  |  | √ |  |  |
| Neiguan (PC6) |  | √ |  |  |  |  |  | √ |  |  |  |  |  | √ | √ |  |  |  |  |  |  |  | √ |  |  |  |  |  |  |
| Jianyu (LI15) |  | √ |  |  | √ |  | √ | √ | √ |  |  | √ |  |  | √ |  |  | √ |  |  |  | √ | √ | √ | √ | √ | √ | √ | √ |
| Jianqian |  | √ |  |  | √ |  | √ | √ | √ |  |  | √ |  | √ | √ |  |  | √ |  |  |  | √ |  |  |  |  |  | √ |  |
| Jianhou |  | √ |  |  |  |  |  | √ |  |  |  | √ |  |  | √ |  |  |  |  |  |  |  |  |  |  |  |  |  |  |
| Chize (LU5) |  | √ |  |  |  |  |  | √ |  |  |  |  |  |  | √ |  |  |  |  |  |  |  | √ |  |  |  |  |  |  |
| Jiquan (HT1) |  | √ |  |  |  |  |  | √ |  |  |  | √ |  | √ | √ |  |  |  |  |  |  |  | √ |  |  |  |  |  |  |
| Zhongwan (CV12) |  |  |  | √ |  |  |  |  |  |  |  |  |  |  |  |  |  |  |  | √ |  |  |  |  |  |  |  | √ |  |
| Guanyuan (CV4) |  |  |  | √ |  |  |  |  |  |  |  |  |  | √ |  |  |  |  |  |  |  |  |  |  |  |  |  |  |  |
| Huaroumen (ST24) |  |  |  | √ |  |  |  |  |  |  |  |  |  |  |  |  |  |  |  |  |  |  |  |  |  |  |  |  |  |
| Wailing (ST26) |  |  |  | √ |  |  |  |  |  |  |  |  |  |  |  |  |  |  |  | √ |  |  |  |  |  |  |  |  |  |
| Shangfengshi point |  |  |  | √ |  |  |  |  |  |  |  |  |  |  |  |  |  |  |  | √ |  |  |  |  |  |  |  |  |  |
| Shangfengshiwai point |  |  |  | √ |  |  |  |  |  |  |  |  |  |  |  |  |  |  |  | √ |  |  |  |  |  |  |  |  |  |
| Binao (LI14) |  |  |  |  | √ |  |  |  |  |  |  | √ |  |  |  |  |  |  |  |  |  |  |  | √ |  | √ |  |  |  |
| Hegu (LI4) |  |  |  |  |  |  |  |  |  |  |  |  |  | √ |  |  |  | √ |  |  |  |  |  |  | √ | √ | √ |  | √ |
| Jianliao (TE14) |  |  |  |  |  | √ | √ |  | √ |  |  |  |  | √ |  | √ | √ | √ |  |  |  |  | √ | √ | √ |  | √ |  |  |
| Zhongzhu (TE3) |  |  |  |  | √ |  |  |  |  |  |  | √ |  |  |  |  |  | √ |  |  |  |  |  |  |  |  |  |  |  |
| Naoshu (SI10) |  |  |  |  |  |  |  |  |  |  |  |  |  |  |  |  |  | √ |  |  |  |  |  |  |  |  |  |  |  |
| Zusanli (ST36) |  |  |  |  |  |  |  |  |  |  |  |  |  | √ |  |  |  |  |  |  |  |  |  |  |  |  |  |  |  |
| Tianzong (SI11) |  |  |  |  | √ |  | √ |  |  |  |  |  |  |  |  |  |  |  |  |  |  |  |  |  | √ |  |  |  |  |
| Houxi (SI3) |  |  |  |  | √ |  |  |  |  |  |  | √ |  | √ |  |  |  | √ |  |  |  |  |  |  |  |  |  |  |  |
| Five-shu Point |  |  |  |  |  | √ |  |  |  |  |  |  |  |  |  | √ | √ |  |  |  |  |  |  |  |  |  |  |  |  |
| Jianjing (GB21) |  |  |  |  |  | √ |  |  |  |  |  |  |  |  |  | √ | √ |  |  |  |  |  |  |  |  | √ |  |  | √ |
| Jianzhen (SI9) |  |  |  |  |  | √ | √ |  | √ |  |  |  |  | √ |  | √ | √ |  |  |  |  | √ |  | √ | √ | √ |  | √ |  |
| Dingpangxian II (MS9) |  |  |  |  |  |  | √ |  |  |  |  |  |  |  |  |  |  |  |  |  |  |  |  |  |  |  |  |  |  |
| Quchi (LI11) |  |  |  |  |  |  | √ |  | √ |  |  |  | √ | √ |  |  |  |  |  |  |  | √ | √ | √ | √ |  | √ | √ | √ |
| Waiguan (TE5) |  |  |  |  |  |  | √ |  | √ |  |  |  | √ | √ |  |  |  |  |  |  |  |  | √ | √ | √ |  | √ | √ | √ |
| Shousanli (LI10) |  |  |  |  |  |  |  |  | √ |  |  |  |  |  |  |  |  |  |  |  |  |  |  | √ | √ | √ |  |  |  |
| Xitong |  |  |  |  |  |  |  |  |  | √ |  |  |  |  |  |  |  |  |  |  |  |  |  |  |  |  |  |  |  |
| Wan 4 |  |  |  |  |  |  |  |  |  |  | √ |  |  |  |  |  |  |  |  |  |  |  |  |  |  |  |  |  |  |
| Wan 5 |  |  |  |  |  |  |  |  |  |  | √ |  |  |  |  |  |  |  |  |  |  |  |  |  |  |  |  |  |  |
| Baihui (GV20) |  |  |  |  |  |  |  |  |  |  |  |  | √ |  |  |  |  |  |  |  |  |  | √ |  |  |  |  |  |  |
| Yintang (EX-HN3) |  |  |  |  |  |  |  |  |  |  |  |  | √ |  |  |  |  |  |  |  |  |  | √ |  |  |  |  |  |  |
| Shenting (GV24) |  |  |  |  |  |  |  |  |  |  |  |  | √ |  |  |  |  |  |  |  |  |  | √ |  |  |  |  |  |  |
| Shuigou (GV26) |  |  |  |  |  |  |  |  |  |  |  |  |  | √ |  |  |  |  |  |  |  |  |  |  |  |  |  |  |  |
| Sanyinjiao (SP6) |  |  |  |  |  |  |  |  |  |  |  |  |  | √ |  |  |  |  |  |  |  |  |  |  |  |  |  |  |  |
| Tongli (HT5) |  |  |  |  |  |  |  |  |  |  |  |  |  | √ |  |  |  |  |  |  |  |  |  |  |  |  |  |  |  |
| Yongquan (KI1) |  |  |  |  |  |  |  |  |  |  |  |  |  | √ |  |  |  |  |  |  |  |  |  |  |  |  |  |  |  |
| Lieque (LU7) |  |  |  |  |  |  |  |  |  |  |  |  |  | √ |  |  |  |  |  |  |  |  |  |  |  |  |  |  |  |
| Yuji (LU10) |  |  |  |  |  |  |  |  |  |  |  |  |  |  |  |  |  | √ |  |  |  |  |  |  |  |  |  |  |  |
| Shangqu (KI17) |  |  |  |  |  |  |  |  |  |  |  |  |  |  |  |  |  |  |  | √ |  |  |  |  |  |  |  |  |  |
| Upper 4 |  |  |  |  |  |  |  |  |  |  |  |  |  |  |  |  |  |  |  |  | √ |  |  |  |  |  |  |  |  |
| Upper 5 |  |  |  |  |  |  |  |  |  |  |  |  |  |  |  |  |  |  |  |  | √ |  |  |  |  |  |  |  |  |
| Upper 6 |  |  |  |  |  |  |  |  |  |  |  |  |  |  |  |  |  |  |  |  | √ |  |  |  |  |  |  |  |  |
| Biliao |  |  |  |  |  |  |  |  |  |  |  |  |  |  |  |  |  |  |  |  |  | √ |  |  |  |  |  |  |  |
| Shuifen (CV9) |  |  |  |  |  |  |  |  |  |  |  |  |  |  |  |  |  |  |  |  |  |  |  |  |  |  |  | √ |  |
| Yinlingquan (SP9) |  |  |  |  |  |  |  |  |  |  |  |  |  |  |  |  |  |  |  |  |  |  |  |  |  |  |  | √ |  |
| Fenglong (ST40) |  |  |  |  |  |  |  |  |  |  |  |  |  |  |  |  |  |  |  |  |  |  |  |  |  |  |  | √ |  |
| Yangchi (TE4) |  |  |  |  |  |  |  |  |  |  |  |  |  |  |  |  |  |  |  |  |  |  |  |  |  |  |  |  | √ |
| Wangu (GB12) |  |  |  |  |  |  |  |  |  |  |  |  |  |  |  |  |  |  |  |  |  |  |  |  |  |  |  |  | √ |
