# Supplementary material for: Effects of Acupuncture on the Recovery Outcomes of Stroke Survivors with Shoulder Pain: A Systematic Review
Source: Front Neurol. 2018 Jan 31;9:30. doi: 10.3389/fneur.2018.00030 (PMC5797784; doi:10.3389/fneur.2018.00030)
Supplement: Supplementary file 5 [file Data_Sheet_5.DOCX]

**Supplementary Data 5: Risk of bias assessment**

| Studies | Was true randomization used for assignment of participants to treatment groups? | Was allocation to treatment groups concealed? | Were treatment groups similar at the baseline? | Were participants blind to treatment assignment?* | Were those delivering treatment blind to treatment assignment?* | Were outcomes assessors blind to treatment assignment? | Were treatments groups treated identically other than the intervention of interest? | Was follow-up complete, and if not, were strategies to address incomplete follow-up utilized? | Were participants analyzed in the groups to which they were randomized? | Were outcomes measured in the same way for treatment groups? | Were outcomes measured in a reliable way? | Was appropriate statistical analysis used? | Was the trial design appropriate, and any deviations from the standard RCT design (individual randomization, parallel groups) accounted for in the conduct and analysis of the trial? |
| --- | --- | --- | --- | --- | --- | --- | --- | --- | --- | --- | --- | --- | --- |
| Huang et al., 2017 | Y | U | Y | NA | NA | U | Y | Y | Y | Y | U | Y | Y |
| Wu et al., 2017 | Y | U | Y | NA | NA | U | Y | Y | Y | Y | U | Y | Y |
| Chen, 2016 | U | U | Y | NA | NA | U | Y | U | U | Y | U | Y | Y |
| He & Gao, 2016 | Y | U | Y | NA | NA | U | Y | N | N | Y | U | Y | Y |
| Tang et al., 2016 | Y | U | Y | NA | NA | U | Y | Y | Y | Y | U | Y | Y |
| Wu et al., 2016 | U | U | Y | NA | NA | U | Y | Y | Y | Y | U | Y | Y |
| Zhou & Chen, 2016 | Y | U | Y | NA | NA | U | Y | U | Y | Y | U | Y | Y |
| Zhong et al., 2016 | Y | U | Y | NA | NA | U | Y | U | Y | Y | U | Y | Y |
| Chen et al., 2015 | Y | U | Y | NA | NA | U | Y | Y | Y | Y | U | Y | Y |
| Li, 2015 | Y | U | Y | NA | NA | U | Y | Y | Y | Y | U | Y | Y |
| Wu et al., 2015 | Y | U | Y | NA | NA | U | Y | Y | Y | Y | U | Y | Y |
| Xu et al., 2015 | Y | U | Y | NA | NA | U | Y | Y | Y | Y | U | Y | Y |
| Zhang & Lu, 2015 | Y | U | Y | NA | NA | U | Y | Y | Y | Y | U | Y | Y |
| Zhang & Zhang, 2015 | U | U | Y | NA | NA | U | Y | U | Y | Y | U | Y | Y |
| Lin et al., 2014 | U | U | Y | NA | NA | U | Y | Y | Y | Y | U | Y | Y |
| Han et al., 2013, 2012, 2011 | U | U | Y | NA | NA | U | Y | Y | Y | Y | U | Y | Y |
| Yang et al., 2011 | U | U | Y | NA | NA | U | Y | Y | Y | Y | U | Y | Y |
| Sun et al., 2012 | Y | U | Y | NA | NA | U | Y | Y | Y | Y | U | Y | Y |
| Zhang et al., 2012 | Y | U | Y | NA | NA | U | Y | U | U | Y | U | Y | Y |
| Chen et al., 2011 | Y | Y | Y | NA | NA | U | Y | U | U | Y | U | Y | Y |
| Shi & Tang, 2011 | Y | U | Y | NA | NA | U | Y | U | U | Y | U | Y | Y |
| Bo et al., 2013 | U | U | Y | NA | NA | U | Y | Y | Y | Y | U | Y | Y |
| Jia et al., 2012 | Y | U | Y | NA | NA | U | Y | Y | Y | Y | U | Y | Y |
| Bao et al., 2012, 2011 | Y | U | Y | NA | NA | U | Y | N | N | Y | U | Y | Y |
| Hong et al., 2011 | Y | U | Y | NA | NA | U | Y | Y | Y | Y | U | Y | Y |
| Yang et al., 2009 | U | U | Y | NA | NA | U | Y | Y | Y | Y | U | Y | Y |
| Xu et al., 2016 | Y | U | Y | NA | NA | U | Y | Y | Y | Y | U | Y | Y |
| Wang & Wang, 2011 | Y | U | Y | NA | NA | U | Y | Y | Y | Y | U | Y | Y |
| Nie & Zhao, 2011 | U | U | Y | NA | NA | U | Y | Y | Y | Y | U | Y | Y |
| No. of studies with low risk of bias (29 studies in total) | 20 | 1 | 29 | NA | NA | 0 | 29 | 20 | 23 | 29 | 0 | 29 | 29 |

Note: Y = Yes; N = No; U = Unclear; NA = Not applicable.

*: Due to the nature of the interventions, study participants and intervention deliverers could easily identify which group the participants were in.

**Reference:**

Joanna Briggs Institute. Joanna Briggs Institute Reviewers’ Manual: 2016 Ed. Australia: Joanna Briggs Institute; 2016.
