# Supplementary material for: Effects of Acupuncture on the Recovery Outcomes of Stroke Survivors with Shoulder Pain: A Systematic Review
Source: Front Neurol. 2018 Jan 31;9:30. doi: 10.3389/fneur.2018.00030 (PMC5797784; doi:10.3389/fneur.2018.00030)
Supplement: Supplementary file 6 [file Data_Sheet_6.DOCX]

**Supplementary Data 6: Results of individual studies on shoulder pain (n=26)**

| Author year | Intervention type | Test or model used | Measure of effects*  (post-intervention values, unless otherwise specified) | p value |
| --- | --- | --- | --- | --- |
| Wu et al. 2017 | Conventional acupuncture | Independent sample t-test | IG: 17.69±7.86  CG: 29.83±6.36 | 0.000 |
| Chen 2016 | Conventional acupuncture | Independent sample t-test | IG: 1.21±1.02  CG: 3.28±0.96 | <0.05 |
| He & Gao 2016 | Conventional acupuncture | Independent sample t-test | IG: 2.00±1.02  CG: 4.00±2.13 | <0.01 |
| Tang et al. 2016 | Conventional acupuncture | Independent sample t-test | IG: 3.41±1.27  CG: 4.85±1.53 | <0.01 |
| Wu et al. 2016 | Conventional acupuncture | Independent sample t-test | IG: -2.11±-2.11^#^  CG: -2.35±0.94 | >0.05 |
| Zhou & Chen 2016 | Conventional acupuncture | Independent sample t-test | IG: 3.49±0.75  CG: 4.08±0.73 | 0.049 |
| Zhong et al. 2016 | Conventional acupuncture | Independent sample t-test | IG: 18.33±12.93  CG: 29.08±12.41 | <0.01 |
| Chen et al. 2015 | Conventional acupuncture | Independent sample t-test | IG: 1.46±0.48  CG: 2.37±0.71 | <0.05 |
| Li 2015 | Conventional acupuncture | Independent sample t-test | IG: 1.53±0.86  CG: 2.37±0.97 | <0.05 |
| Wu et al. 2015 | Conventional acupuncture | Independent sample t-test | IG: 2.55±1.08  CG: 4.50±1.19 | <0.01 |
| Xu et al. 2015 | Conventional acupuncture | Independent sample t-test | IG: 3.52±1.45  CG: 4.86±1.67 | <0.01 |
| Zhang & Lu 2015 | Conventional acupuncture | Independent sample t-test | IG: 2.49±1.77  CG: 3.55±1.66 | <0.05 |
| Zhang & Zhang 2015 | Conventional acupuncture | Independent sample t-test | IG: 3.81±0.96  CG: 4.32±1.46 | 0.001 |
| Lin et al. 2014 | Conventional acupuncture | Independent sample t-test | IG: 19.43±11.82  CG: 28.17±12.27 | <0.05 |
| Yang et al. 2011 | Conventional acupuncture | Independent sample t-test | IG: 2.56±0.76  CG: 4.52±0.85 | <0.05 |
| Sun et al. 2012 | Conventional acupuncture | Independent sample t-test | IG: 4.23±1.92  CG: 5.40±2.28 | <0.05 |
| Zhang et al. 2012 | Conventional acupuncture | Independent sample t-test | IG: 1.11±0.98  CG: 3.40±1.52 | <0.05 |
| Chen et al. 2011 | Conventional acupuncture | Independent sample t-test | IG: 2.69±1.29  CG: 4.45±1.66 | <0.05 |
| Shi & Tang 2011 | Conventional acupuncture | Independent sample t-test | Immediate post-intervention:  IG: 2.97^$^  CG: 4.52  1-month follow-up:  IG: 4.00^$^  CG:5.2 | 0.01  0.03 |
| Bo et al. 2013 | Electro-acupuncture | Independent sample t-test | IG: 33.12±5.34  CG: 41.53±6.41 | <0.05 |
| Jia et al. 2012 | Electro-acupuncture | Independent sample t-test | IG: 2.48±1.81  CG: 3.56±1.87 | <0.05 |
| Bao et al. 2012 | Electro-acupuncture | Independent sample t-test | IG: 2.83±2.24  CG: 3.95±2.31 | <0.05 |
| Hong et al. 2011 | Electro-acupuncture | Independent sample t-test | IG: 2.01±1.50  CG: 4.25±1.54 | <0.01 |
| Xu et al. 2016 | Fire needle acupuncture | Independent sample t-test | IG: 2.29±0.39  CG: 3.94±0.55 | <0.05 |
| Wang & Wang 2011 | Fire needle acupuncture | Independent sample t-test | IG: 2.21±1.32  CG: 5.02±2.12 | <0.05 |
| Nie & Zhao 2011 | Warm acupuncture | Independent sample t-test | IG: 1.38±0.43  CG: 2.78±0.56 | <0.01 |

IG: intervention group

CG: control group

*: lower value indicates lower level/greater reduction in level of shoulder pain

^#^: value of change from baseline to follow-up

^$^: standard deviation not reported
