# Supplementary material for: Effects of Acupuncture on the Recovery Outcomes of Stroke Survivors with Shoulder Pain: A Systematic Review
Source: Front Neurol. 2018 Jan 31;9:30. doi: 10.3389/fneur.2018.00030 (PMC5797784; doi:10.3389/fneur.2018.00030)
Supplement: Supplementary file 7 [file Data_Sheet_7.DOCX]

**Supplementary Data 7: Results of individual studies on upper extremity function (n=27)**

| Author year | Intervention type | Test or model used | Measure of effects*  (post-intervention values, unless otherwise specified) | p value |
| --- | --- | --- | --- | --- |
| Huang et al. 2017 | Conventional acupuncture | Independent sample t-test | IG: 3.50±1.55^#^  CG: 2.23±1.63 | <0.01 |
| Wu et al. 2017 | Conventional acupuncture | Independent sample t-test | IG: 55.82±10.36  CG: 40.33±9.88 | 0.000 |
| Chen 2016 | Conventional acupuncture | Independent sample t-test | IG: 23.14±5.28  CG: 15.86±4.57 | <0.05 |
| He & Gao 2016 | Conventional acupuncture | Independent sample t-test | IG: 42.17±12.05  CG: 34.41±11.23 | <0.05 |
| Tang et al. 2016 | Conventional acupuncture | Independent sample t-test | IG: 53.92±6.84  CG: 48.79±7.36 | <0.05 |
| Wu et al. 2016 | Conventional acupuncture | Independent sample t-test | IG: 12.56±5.31^#^  CG: 9.32±3.78 | >0.05 |
| Zhong et al. 2016 | Conventional acupuncture | Independent sample t-test | IG: 53.11±9.32  CG: 42.35±8.49 | 0.00 |
| Chen et al. 2015 | Conventional acupuncture | Independent sample t-test | IG: 39.27±7.14  CG: 30.56±5.22 | <0.05 |
| Li 2015 | Conventional acupuncture | Independent sample t-test | IG: 47.57±6.19  CG: 39.16±6.08 | <0.05 |
| Wu et al. 2015 | Conventional acupuncture | Independent sample t-test | IG: 25.28±10.01  CG: 21.97±8.32 | <0.01 |
| Xu et al. 2015 | Conventional acupuncture | Independent sample t-test | IG: 53.27±6.65  CG: 49.26±7.92 | <0.05 |
| Zhang & Lu 2015 | Conventional acupuncture | Independent sample t-test | IG: 36.66±9.46  CG: 31.19±8.88 | <0.05 |
| Lin et al. 2014 | Conventional acupuncture | Independent sample t-test | IG: 54.87±9.79  CG: 43.26±9.38 | <0.05 |
| Han et al. 2011 | Conventional acupuncture | Independent sample t-test | IG: 49.84±13.52  CG: 41.62±13.55 | <0.05 |
| Yang et al. 2011 | Conventional acupuncture | Independent sample t-test | IG: 49.84±13.52  CG: 41.62±10.55 | <0.05 |
| Sun et al. 2012 | Conventional acupuncture | Independent sample t-test | IG: 31.52±6.40  CG: 27.65±7.36 | <0.05 |
| Zhang et al. 2012 | Conventional acupuncture | Independent sample t-test | IG: 22.46±5.61  CG: 16.22±4.18 | <0.05 |
| Chen et al. 2011 | Conventional acupuncture | Independent sample t-test | IG: 24.13±25.48  CG: 14.73±10.29 | <0.05 |
| Shi & Tang 2011 | Conventional acupuncture | Independent sample t-test | Immediate post-intervention:  IG: 42.15±21.34  CG: 35.67±19.28  1-month follow-up  IG: 40.56±22.14  CG: 30.12±18.23 | 0.001  0.002 |
| Bo et al. 2013 | Electro-acupuncture | Independent sample t-test | IG: 35.39±6.57  CG: 27.45±6.42 | <0.05 |
| Jia et al. 2012 | Electro-acupuncture | Independent sample t-test | IG: 36.77±14.58  CG: 31.21±15.97 | <0.05 |
| Bao et al. 2011 | Electro-acupuncture | Independent sample t-test | IG: 36.46±13.55  CG: 28.47±14.02 | <0.05 |
| Hong et al. 2011 | Electro-acupuncture | Independent sample t-test | IG: 61.89±8.64  CG: 52.32±9.47 | <0.05 |
| Yang et al. 2009 | Electro-acupuncture | Independent sample t-test | IG: 22.26±4.31^#^  CG: 12.00±3.13 | 0.000 |
| Xu et al. 2016 | Fire needle acupuncture | Independent sample t-test | IG: 38.92±6.36  CG: 30.12±5.89 | <0.05 |
| Wang & Wang 2011 | Fire needle acupuncture | Independent sample t-test | IG: 18.34±8.53  CG: 11.65±4.37 | <0.01 |
| Nie & Zhao 2011 | Warm acupuncture | Independent sample t-test | IG: 45.34±12.21  CG: 38.54±13.67 | <0.05 |

IG: intervention group

CG: control group

*: higher value indicates better/greater improvement in upper extremity function

^#^: value of change from baseline to follow-up
