# Supplementary material for: Effects of Acupuncture on the Recovery Outcomes of Stroke Survivors with Shoulder Pain: A Systematic Review
Source: Front Neurol. 2018 Jan 31;9:30. doi: 10.3389/fneur.2018.00030 (PMC5797784; doi:10.3389/fneur.2018.00030)
Supplement: Supplementary file 8 [file Data_Sheet_8.DOCX]

**Supplementary Data 8: Results of individual studies on edema (n=6)**

| Author year | Intervention type | Test or model used | Measure of effects*  (post-intervention values, unless otherwise specified) | p value |
| --- | --- | --- | --- | --- |
| Huang et al. 2017 | Conventional acupuncture | Independent sample t-test | IG: 10.81±1.73  CG: 15.62±1.30 | <0.01 |
| Tang et al. 2016 | Conventional acupuncture | Independent sample t-test | IG: 1.73±1.12  CG: 2.48±1.36 | <0.05 |
| Xu et al. 2015 | Conventional acupuncture | Independent sample t-test | IG: 1.02±0.44  CG: 1.46±0.58 | <0.05 |
| Hong et al. 2011 | Electro-acupuncture | Independent sample t-test | IG: 1.15±1.01  CG: 2.53±1.27 | <0.05 |
| Wang & Wang 2011 | Fire needle acupuncture | Independent sample t-test | IG: 1.03±0.91  CG: 3.32±1.40 | <0.05 |
| Nie & Zhao 2011 | Warm acupuncture | Independent sample t-test | IG: 1.22±0.21  CG: 2.56±0.67 | <0.01 |

IG: intervention group

CG: control group

*: lower value indicates lower level of edema
