# Supplementary material for: Effects of Acupuncture on the Recovery Outcomes of Stroke Survivors with Shoulder Pain: A Systematic Review
Source: Front Neurol. 2018 Jan 31;9:30. doi: 10.3389/fneur.2018.00030 (PMC5797784; doi:10.3389/fneur.2018.00030)
Supplement: Supplementary file 9 [file Data_Sheet_9.DOCX]

**Supplementary Data 9: Results of individual studies on range of motion of the affected shoulder (n=2)**

| Author year | Intervention type | Test or model used | Measure of effects  (post-intervention values, unless otherwise specified) | p value |
| --- | --- | --- | --- | --- |
| Han et al. 2012 | Conventional acupuncture | Independent sample t-test | IG: 0.42±0.66^  CG: 1.13±0.49 | <0.05 |
| Bao et al. 2012 | Electro-acupuncture | Independent sample t-test | IG: 11.57±3.25*  CG: 9.43±3.63 | <0.05 |

IG: intervention group

CG: control group

^^^: lower value indicates better range of motion

*: higher value indicates better range of motion
