# Supplementary material for: Effects of Acupuncture on the Recovery Outcomes of Stroke Survivors with Shoulder Pain: A Systematic Review
Source: Front Neurol. 2018 Jan 31;9:30. doi: 10.3389/fneur.2018.00030 (PMC5797784; doi:10.3389/fneur.2018.00030)
Supplement: Supplementary file 10 [file Data_Sheet_10.DOCX]

**Supplementary Data 10: Results of individual studies on physical function (n=7)**

| Author year | Intervention type | Test or model used | Measure of effects*  (post-intervention values, unless otherwise specified) | p value |
| --- | --- | --- | --- | --- |
| He & Gao 2016 | Conventional acupuncture | Independent sample t-test | IG: 68.32±22.20  CG: 47.11±19.37 | <0.05 |
| Chen et al. 2015 | Conventional acupuncture | Independent sample t-test | IG: 56.41±19.72  CG: 53.01±20.17 | >0.05 |
| Han et al. 2013 | Conventional acupuncture | Independent sample t-test | IG: 82.59±15.14  CG: 70.49±13.29 | <0.05 |
| Chen et al. 2011 | Conventional acupuncture | Independent sample t-test | IG: 64.73±27.21  CG: 51.33±21.53 | <0.05 |
| Shi & Tang 2011 | Conventional acupuncture | Independent sample t-test | Immediate post-intervention:  IG: 63.47±18.22  CG: 55.25±20.33  1 month post treatment regimen:  IG: 60.21±24.55  CG: 48.46±22.56 | 0.001  0.001 |
| Bao et al. 2012 | Electro-acupuncture | Independent sample t-test | IG: 61.41±21.95  CG: 51.79±20.76 | <0.05 |
| Hong et al 2011 | Electro-acupuncture | Independent sample t-test | IG: 62.25±6.88  CG: 52.12±7.55 | <0.01 |

IG: intervention group

CG: control group

*: higher value indicates better physical function
